# Design, synthesis and biological evaluation on *N*- heteroaryl compounds as probable NNRTIs against laboratory adapted strains and the primary isolates of HIV-1

**DOI:** 10.1186/1471-2334-14-S3-E15

**Published:** 2014-05-27

**Authors:** Anuradha Singh, Madhu Yadav, Ritika Srivastava, Nidhi Singh, Ashwini Godse, Rahul Suryawanshi, Ashwini Dhamanage, Smita Kulkarni, Ramendra K Singh

**Affiliations:** 1 Nucleic Acids & Antiviral Research Laboratory, Department of Chemistry, University of Allahabad, India; 2 National AIDS Research Institute, Pune, India

## Background

The emergence of drug resistant HIV strains and suitability of NNRTIs as potent anti-HIV molecules used in HAART did attract our attention towards developing NNRTIs. We, therefore, focused on developing some new NNRTIs utilizing benzimidazole and pyrimidine moieties to match the hydrophobic nature of the allosteric pocket in HIV-RT.

## Methods

Compounds designed on the basis of extensive computational studies, were finally synthesized through facile synthetic route and characterized using various chromatographic and spectral techniques. All synthesized molecules have been screened against HIV-1 using TZM bl assay and laboratory adapted strains HIV-1 IIIB (X4, subtype B), HIV-1 Ada5 (R5, Subtype B) and the primary isolates HIV-1UG070 (X4, Subtype D).

## Results

Cell based assay showed that majority of the compounds were active at micro molar concentrations (1.39-17.39 µM) and the SI value ranged between 10.77-17.39 against lab adapted strains and 5.8–13.91 against primary isolates. The studies on structure–activity relationship were also consistent with the experimental data.

## Conclusions

In view of the results obtained, these compounds may be developed as potent inhibitors of HIV-1 replication with suitable structural/pharmacophore modifications.

